# Removal of Chromium (III) and Reduction in Toxicity in a Primary Tannery Effluent Using Two Floating Macrophytes

**DOI:** 10.3390/toxics12020152

**Published:** 2024-02-16

**Authors:** Tomás R. López Arias, Deidamia Franco, Leonida Medina, César Benítez, Verónica Villagra, Shaun McGahan, Giselle Mariza Duré, Hajime G. Kurita-Oyamada

**Affiliations:** 1 Grupo de Investigación en Biotecnología Ambiental, Departamento de Biotecnología, Facultad de Ciencias Exactas y Naturales, Universidad Nacional de Asunción, San Lorenzo 1039-1804, Paraguay; profedeidy@gmail.com (D.F.); cesaritobt@gmail.com (C.B.); smcgahan@facen.una.py (S.M.); giselledure@facen.una.py (G.M.D.); guillermo.kurita@facenuna.edu.py (H.G.K.-O.); 2 Instituto Nacional de Tecnología, Normalización y Metrología (INTN), Asunción 1518, Paraguay; lmedina@intn.gov.py; 3 Central Public Health Laboratory, Ministry of Health and Social Welfare, Asunción 1429, Paraguay; v.villagra2@gmail.com

**Keywords:** tannery wastewater, chromium, *Eichhornia crassipes*, *Salvinia auriculata*, bioremediation, toxicity, *Daphnia magna*, *Danio rerio*

## Abstract

Trivalent chromium (Cr(III)) is a contaminant with toxic activity. Its presence in waters and soils is usually related to industrial activities such as tanneries. The aim of this study was to compare the removal of Cr(III) in hydroponic solutions and tannery effluents using two floating macrophytes: *Salvinia auriculata* and *Eichhornia crassipes*. First, to determine the chromium removal capacity in solution and the bioaccumulation factor (BAF) in tissues of each plant, experiments were set up with contaminated solutions with Cr(III) concentrations of 2, 5, 10, 20, and 40 mg/L. Subsequently, both plant species were exposed to a primary tannery effluent contaminated with 12 mg/L of Cr(III) in order to study the removal capacity of organic and inorganic matter, as well as the acute toxicity in the water flea (*Daphnia magna*) and genotoxicity in zebrafish (*Danio rerio*). Tests carried out on nutrient solutions revealed that both plants have a high capacity for removing Cr(III) in solution. The BAF in tissues was higher in *E. crassipes* compared to *S. auriculata*. In the experiments with a tannery effluent, both species presented low nutrient and organic matter removal efficiency, but they showed good Cr(III) removal capacity, with average reduction values of 57% for *S. auriculata* and 54% for *E. crassipes* after 72 h of exposure. *E. crassipes* contributed most to the reduction in acute toxicity in *D. magna*, while *S. auriculata* did not show a similar effect. However, both plant species managed to reduce the genotoxicity marker in *D. rerio* when compared with the initial effluent and the control.

## 1. Introduction

Contamination of the aquatic environment by heavy metals is a serious environmental problem that threatens ecosystems, agriculture, and human health. Metals can accumulate in plants and enter trophic chains, with consequences for human health and potential ecotoxic effects for other biological organisms [1]. Chromium is considered one of the main inorganic water pollutants and can be present in two oxidation states, Cr(III) and Cr(VI). Cr(III) and Cr(VI) can interconvert, generally favoring the reduction in Cr(VI) to Cr(III) in most environmental situations, such as the presence of other metals with redox activity, exposure to light or atmospheric water [2]. Cr(VI) is generally considered more toxic; hence, most studies were conducted with it. On the other hand, several studies have also shown that Cr(III) is a potential genotoxic agent widely used in industrial processes [3,4]. Wastewater from tanneries has become one of the dominant sources of industrial pollution [5] and is routinely identified as the main source of Cr [6]. The most commonly used methods for tanning leather are vegetable (with tannin) and mineral (with trivalent chromium salts). During mineral tanning, leftover Cr compounds can remain in the wastewater, leaving a high content in the effluent [7]. However, many other compounds are also used in the tanning process, totaling 175 different chemicals, such as sodium hydroxide, sodium chloride, pentachlorophenol, sodium sulfide, enzymes, lime slurry, chlorides, sulfuric acid, formic acid, ammonium chloride, ammonium sulfate, metallic salts, and others [8]. When not properly treated, this makes tannery effluents complex and with high potential environmental impact. Cr-contaminated tannery effluents can cause phytotoxic and genotoxic effects in *Allium cepa* [9] and aneugenicity and clastogenicity in the fish *Oreochromis niloticus* [10]. Among the ecotoxicological effects of tannery effluents, we can highlight changes observed in different species of plants and animals exposed to them, such as invertebrates, fish, amphibians, birds, and mammals.

Effective Cr(III) removal is essential but challenging in tanneries. Conventional wastewater treatment technologies often fail to completely remove various types of pollutants from water; they are costly, energy-intensive, and environmentally unfriendly [11]. These problems have led to the search for technologies that can remove pollutants efficiently and, at the same time, are environmentally friendly, which has given a strong impulse to wetland treatment as a viable alternative [12,13]. These biological systems are based on the use of plants and associated microorganisms to stabilize, transfer, degrade, or reduce the concentrations or toxic effects of pollutants [1]. Treatment wetlands are considered a viable alternative for treating tannery effluents in developing countries, using natural processes to remove pollutants from water [5]. Plant selection is an important issue in constructed wetlands, as plants must survive the potentially toxic effects of wastewater and remove contaminants. The phytoremediation potential of plants is generally evaluated by determining the bioaccumulation factor (BAF), described as the ratio between the concentration of contaminants in the parts of the plant and the surrounding environment, and the translocation factor (TF), defined as the ratio of accumulation of the contaminant in the aerial part of the plant compared to the root [14]. In the case of Cr(III), plants take up the metal by simple diffusion at the cation exchange sites of the cell wall, while Cr(VI) can actively enter through sulfate transport channels [15]. Several studies have shown the promising potential of constructed wetlands for the removal of multiple pollutants, including chromium [16,17]. Macrophytes have an enormous potential to accumulate heavy metals, which have been harnessed for the removal of metals from water and soil [18,19].

Regarding the plants studied in this study, *Salvinia auriculata* Aubl. is a floating aquatic fern of lentic waters, native to the Americas, widely distributed in Argentina, Brazil, Paraguay, and Uruguay [20]. In Paraguay, it is very common to find it in the eastern region, along with other species of the genus [21]. Predictive models carried out in a scenario of variations caused by climate change indicate that this species will continue to gain habitats outside its native distribution area [22]. *Eicchornia crassipes* (Mart.) Solms is a perennial floating herb of the family Pontederiaceae native to South America [23]. It develops in lentic and lotic environments with little current, with numerous roots and stoloniferous horizontal stems that favor the formation of very dense masses that can be initiators of *embalsados* (a type of freshwater floating island). In Paraguay, it is found in the humid Chaco region and in the eastern region [21]. It currently has a cosmopolitan distribution and is considered invasive in some regions [24]. Both species can be very invasive, even in their native habitats of Paraguay and Brazil [25]. *Eichhornia crassipes* has a longer history of use in phytoremediation than *Salvinia auriculata* [26,27]. *E. crassipes* can be used to treat urban leachates [28], in cyanide removal [29], and the treatment and removal of chromium in tannery effluents [30]. *S. auriculata* can remove heavy metals in a solution since this macrophyte has the ability to accumulate mercury in roots and tissues [31] and remove Cr(III) and Cr(VI) in hydroponic solutions [32,33]. Other species of the *Salvinia* genus have been extensively studied in metal phytoremediation. *Salvinia minima* can remove Cd(II), Pb(II), and Cr(VI) but is affected by environmental conditions such as pH and light intensity [34]. It has also been shown that dead *S. molesta* tissue functions as a Cr(VI) adsorbent, with an adsorption capacity of 33.33 mg/g following a Langmuir isotherm model [35]. Another study showed that after chemical modification of the surface of *Salvinia* sp., its Cr(VI) adsorption capacity can be improved [36].

Different model organisms can be used to study the toxicity of metals and effluents. Fish species such as *Danio rerio* (zebrafish) have been widely used as biomonitors because they are considered the most suitable models for monitoring the contamination of systems [37,38]. Cr(VI) at concentrations of 2 mg/L induces nuclear abnormalities in erythrocytes in *D. rerio*, which may serve as extremely sensitive endpoints of toxicological stress indicators of aquatic contaminants [39]. Among invertebrates, *Daphnia magna* has been shown to be very sensitive to the toxicity of tannery effluents [40] and thus can be used to evaluate the toxicity removal efficiency of wastewater treatment plant effluents via anaerobic reactors and wetlands [41]. Both *D. rerio* and *D. magna* are organisms widely used in ecotoxicological studies.

The literature review revealed that assays had been performed on *E. crassipes* and *Salvinia* sp. to remove pollutants from wastewater [42,43,44]. Also, its importance lies in their aptitude to be employed in rural areas due to their low consumption of conventional energy and practicality in the assembly and operation of treatment systems [45]. Regarding chromium, most removal studies focus on Cr(VI) despite the fact that Cr(III) is currently widely used in industrial processes. The conventional leather tanning process uses basic chromium sulfate (Cr(OH)SO_4_) [46]. However, few studies have comparatively examined the ability of these species to remove toxicity and genotoxicity in tannery effluents [47]. Most of the tanneries processing leather in Paraguay use basic chromium sulfate (Cr(OH)SO_4_) in the tanning stage, and although there are treatments to reduce the load of toxic compounds, the concentration of chromium in the wastewater at the final outlet of the treatment plant is above the threshold admitted by current legislation [6]. Considering that tanneries are an important source of pollution in Paraguay and other developing countries, the objective of the present work was to compare the removal of Cr(III) in hydroponic solutions, and subsequently in tannery effluents, using two local floating macrophytes: *Salvinia auriculata* and *Eichhornia crassipes*. Finally, to evaluate the performance of the plants with the real effluent, the change in physicochemical parameters and the variation of toxicity were studied using *Daphnia magna* and *Danio rerio* as model organisms.

## 2. Materials and Methods

### 2.1. Plant Specimen Collection, Experimental Conditions of Phytosorption Assays, and Removal of Cr(III)

*Eichhornia crassipes* (Mart.) Solms was obtained from Lake Ypacaraí, in the wetlands area of the city of Ypacaraí, Paraguay (UTM: 21J, X:46,9630.65 m E, Y: 719,4572.38 m S). *Salvinia* aff. *auriculata* Aubl. was collected from the Yrupe Pond, located on the left bank of the Paraguay River, in the city of Asunción, Paraguay (UTM: 21J, 433,344.98 m E, 7200,168.33 m S). The species were taxonomically identified, and their duplicates were deposited in the Herbariums of the Faculty of Exact and Natural Sciences of the National University of Asunción (FACEN-UNA), the Natural History Museum, United Kingdom (BM), and of the Faculty of Chemical Sciences of the National University of Asunción (FCQ-UNA) with the codes 1742 and 1744, respectively (J. De Egea, T. López Arias, 9 May 2017). The plants were acclimatized for 60 days until new seedlings were grown in the greenhouse. Acclimatization was carried out by gradual mixing with water enriched with a commercially formulated nutrient (NPK, 20:20:20) and then with a modified APHA medium [48]. Only seedlings produced and grown in the greenhouse at a mean temperature of 25 °C were used in the experiments.

The ability of *E. crassipes* and *S. auriculata* to remove Cr (III) in solution was evaluated. We diluted the Cr(III) with a 2% HNO_3_ solution. Each species was exposed to 0.5 L of solution at 2, 5, 10, 20, and 40 mg/L. Similar concentrations were used by Singh and Sinha [43] and Woldemichael et al. [30]. Five replicates per treatment were prepared with two controls. The samples were evaluated for 48–54 h. *S.auriculata* was evaluated at 0, 6, 20, 29, and 54 h post-exposure to the solutions mentioned above, and *E crassipes* was evaluated at 0, 3, 6, 15, 24, and 48 h post-exposure to these solutions. The determination of chromium in a solution was direct. To determine chromium in plant tissues, the plants were harvested at the end of the period of the assay, and the ones exposed to concentrations of 2 and 20 mg/L, were selected. A total of 2 mg/L was chosen because it is the limit for discharge into wastewater Res 222/02 of the Ministry of the Environment of Paraguay (MADES) [49] and 20 mg/L because it is the average of all the concentrations studied. The plant samples were washed with tap water and dried at 75 °C for 48 h in an oven. The size of the plants and the ease of separating the root from the aerial part were considered. For *S. auricula*, the bioaccumulation of Cr(III) was studied in the entire plant as a whole, while in *E. crassipes* the root was separated from the aerial part, and the translocation factor was additionally determined. For the analysis of Cr(III) content in the plant, wet extraction and microwave digestion were performed (brand: SINEO, model: Microwave Reaction system TANK Basic, manufacturer: SINEO, city: Shanghai, country: China), for which 0.5 g of the dried plant material samples were weighed and processed according to the methodology of the microwave digester equipment used. 

The bioaccumulation factor (BAF) was determined using Equation (1).

(1) $$\mathrm{BAF}=\frac{\mathrm{mgCr}/\mathrm{Kg}\;\mathrm{body}\;\mathrm{of}\;\mathrm{the}\;\mathrm{plant}\;(\mathrm{dry}\;\mathrm{weight})}{\mathrm{mgCr}/\mathrm{Kg}\;\mathrm{in}\;\mathrm{external}\;\mathrm{solution}}$$

The translocation factor (TF), understood as the capacity of the plant to mobilize the element from the roots to the leaves, was estimated only for *E. crassipes* through Equation (2)

(2) $$\mathrm{TF}=\frac{\mathrm{mgCr}/\mathrm{Kg}\;\mathrm{accumulated}\;\mathrm{in}\;\mathrm{aerial}\;\mathrm{part}\;(\mathrm{dry}\;\mathrm{weight})}{\mathrm{mgCr}/\mathrm{Kg}\;\mathrm{accumulated}\;\mathrm{in}\;\mathrm{root}\;(\mathrm{dry}\;\mathrm{weight})}$$

### 2.2. Phytoremediation of Tannery Effluent in Floating Wetlands with E. crassipes and S. auriculata

A primary effluent treated by coagulation–flocculation from a leather tanning company in the city of Asunción was used. Previous analyses of the effluent yielded values < 1 mg Cr(III)/L; therefore, the effluent was contaminated with 12.34 mg Cr(III)/L for the tests. The assays were conducted in triplicate with 40 L of the effluent in systems of 0.63 m length, 0.44 m width, and 0.15 m depth of liquid. The reactors were operated discontinuously for 72 h without aeration and with manual agitation every 6 h. The treatments were performed in triplicate for each plant, with a control without macrophytes. Samples of the effluent were taken from each replicate at the beginning and the end of the exposure period. The collected and filtered samples were kept in 10 mL falcon tubes and refrigerated until analysis. The samples obtained at the end of the study were used to determine chromium, in addition to the main physicochemical parameters, and the toxicity levels measured with *D. magna* and *D. rerio*.

### 2.3. Chromium Analysis and Physicochemical Parameters of the Tannery Primary Effluent

Chromium was quantified using atomic absorption spectrophotometry measurements (brand: SHIMADZU, model: AA-7800, manufacturer: Shimadzu Inc. city: Kyoto, country: Japan) according to the standardized methodology, Air-Acetylene Flame Method 3111-B [50]. Hexavalent chromium was determined at the beginning and end of the experiments to observe if trivalent chromium was oxidized, according to the Colorimetric Method 3500-Cr D (APHA, 2012). The drying and extraction of chromium from the plant material and its quantification were performed in the Environmental Testing Department of the National Institute of Technology, Standardization and Metrology (INTN) laboratory. The physicochemical tests of the primary effluent were carried out at the Effluent Laboratory of FACEN. The wastewater exhibited the following characteristics: five-day biological oxygen demand (BOD) (456 mg/L), chemical oxygen demand (COD), (657 mg/L), BOD/COD ratio: 0.69, total phosphorus (0.65 mg/L), N-ammonia (60.11 mg/L N-NH_4_), total Kjeldhal nitrogen (TKN) (82.5 mg/L), nitrite (1.99 mg/L), nitrate (<0.010 mg/L), fats and oils (<0.5 mg/L), sulfurs (22.3 mg/L), pH (8.5 UpH), dissolved oxygen (DO) (0.8 mg/L), color (150 Pt/Co), total suspended solids (TSS) (40 mg/L), volatile suspended solids (VSS) (33 mg/L), total sedimentable solids (SS) (<0.010 mg/L), cadmium (<0.001 mg/L), cyanide (<0.002 mg/L), copper (0.056 mg/L), and soluble iron (0.23 mg/L).

The removal efficiency (R%) was determined using Equation (3):

(3) $$R(\%)=\left[\left(\mathrm{Ci}-\mathrm{Cf}\right)/\mathrm{Ci}\right]\;\times \;100$$

where R is the pollutant reduction, Ci is the initial pollutant concentration, and Cf is the final pollutant concentration [51]. 

### 2.4. Bioassays 

#### 2.4.1. Toxicity Assay with *Daphnia magna*

The effect of tannery effluent on aquatic invertebrates was evaluated using *D. magna*, a commonly used ecotoxicity test species. The organisms used were from hard reconstituted water-based culture batches [50] fed with *Chlorella* sp. and maintained in the Laboratory of Environmental Biotechnology of FACEN-UNA. Maintenance and testing were performed according to the recommendations of the OECD Test Guideline [52]. Samples of untreated wastewater and those treated with *E. crassipes* and *S. auriculata* were tested. The count of immobile individuals was performed 48 h after the start of the tests, and the effective concentration 50 (EC_50_) was selected as the endpoint measurement. EC_50_ was calculated following the log-probit method. The acute toxicity unit (ATu) was expressed as (100/EC_50_).

#### 2.4.2. Micronucleus Test in *Danio rerio*

Experiments were performed at sublethal concentrations (12.5% *v*/*v*) following the methodology proposed by Bolognesi [37]. At least eight individuals were exposed per concentration in containers with 15 L of the samples diluted in dechlorinated drinking water for 14 days, without food, with a photoperiod of 16 h of light and 24–26 °C temperature. The tanks were aerated, and pH, DO, and temperature were monitored. After the time had elapsed, the fish were sacrificed by freezing, and peripheral blood was obtained from the gills with heparinized pipette tips. Staining was performed using Schiff’s reagent technique. Two thousand erythrocytes per fish were randomly analyzed. Toxicity was determined using micronucleus analysis. The analysis of the preparations was performed under the microscope at 1000× magnification, using the objective with immersion oil.

#### 2.4.3. Statistical Data Analysis

The data analyses were performed using Microsoft Excel and SPSS 21.0. Normality was tested using the Shapiro–Wilk test, and homoscedasticity was checked with Levene’s test. Student’s *t*-test was applied to check the differences between the final removal capacities of Cr(III) in synthetic solution for each plant. A level of *p* < 0.05 was used in all comparisons. A one-way analysis of variance (*p* < 0.05) was used to investigate a statistically significant difference in the mean removal efficiencies of pollutants between the constructed wetland units exposed to tannery effluent. Multiple comparisons were performed using Tukey’s HSD tests. Statistical analyses were performed with SPSS Statistics 21.0.

## 3. Results and Discussion

### 3.1. Removal and Accumulation of Chromium (III) in Nutrient Solution and BAF

**Figure 1:**
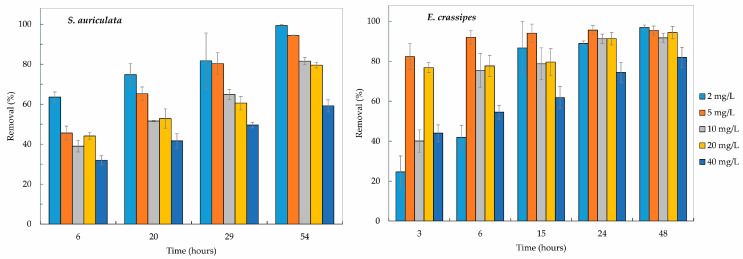
Removal of Cr(III) from two macrophytes exposed to synthetic solution.

The removal capacity of Cr(III) from a solution was characterized by exposing the two macrophyte species to concentrations ranging from 2 to 40 mg/L for 48 to 54 h. *S. auriculata* was evaluated at 0, 6, 20, 29, and 54 h post-exposure to the solutions mentioned above, and *E crassipes* was evaluated at 0, 3, 6, 15, 24, and 48 h post-exposure. The results of Cr(III) removal are presented in Figure 1.

**Figure 2:**
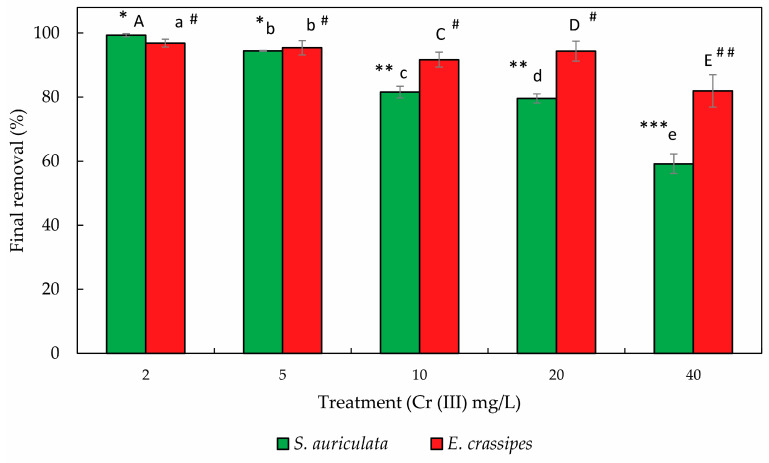
Final removal at different Cr(III) concentrations for each plant. Each value is the mean of the five replicates, and the bars represent standard deviations. Uppercase and lowercase letters indicate significant differences between species for each treatment obtained using Student’s *t*-test (*p* < 0.05). The symbols correspond to the ANOVA and the homogeneous subsets, where the asterisk (*) corresponds to *S. auriculata* and the pound (#) to *E. crassipes* (*p* < 0.05). More than one asterisk or pound sign indicates significant differences between treatment subgroups for each species.

The final removal in *S. auriculata* was 99.3 ± 0.48%, 94.4 ± 0.15%, 81.6 ± 1.8%, 79.6 ± 1.41%, and 59 ± 3.03% for the treatments at 2, 5, 10, 20, and 40 mg/L Cr(III) in a hydroponic solution, while *E. crassipes* was able to remove 96.9 ± 0.01%, 95.4 ± 0.33%, 91.7 ± 2.07%, 94.3 ± 4.4%, and 81.9 ± 15.16% for the treatments at the same concentrations (Figure 1 and Figure 2). *E. crassipes* was more efficient for Cr(III) reduction in a solution for high concentrations (5, 10, 20, and 40 mg/L), with final removals significantly higher than *S. auriculata* (*p* < 0.05). However, *S. auriculata* presented the best results in treatments at the lowest concentration (2 mg/L) of Cr(III) (*p* < 0.05). As the concentration of chromium in the treatments increased, a reduction in individual and overall removal efficiency was observed for both plants (Figure 2). This significant reduction (*p* < 0.05) is observed in *S. auriculata* in the treatments at 10 and 40 mg/L of Cr(III), while in *E. crassipes* a reduction is observed only at the highest concentration (40 mg/L).

Both macrophytes showed Cr(III) removal values above 40%. The highest removal was observed in the first 20 to 24 h of the experiment, although neither of the two plants managed to remove 100% of the Cr(III). These results of rapid removal of Cr(III) in the first hours of exposure are similar to previous studies [53,54]. Maine et al. propose that Cr(III) removal kinetics involves two processes or components: fast and slow [55]. The fast component occurs during the first hours of contact and is responsible for most of the Cr(III) removal from water, where physical adsorption is the most important removal mechanism. The slow component would be explained via the precipitation and cellular absorption of chromium.

Tissue metal accumulation increased with Cr(III) concentrations (Table 1). Of the two macrophytes studied, *E. crassipes* showed a higher BAF, mainly in the root (Table 2). The metal accumulation values in the tissue of *S. auriculata* measured in this study are lower than those reported by Espinoza-Quiñones et al., who exposed *S. auriculata* to concentrations of 5 mg/L Cr(III) in a hydroponic solution, obtaining an accumulation of 10.12 and 2.08 mg/g in root and leaf tissue, respectively, after 7 days [32]. Conversely, Ponce et al. measured Cr(III) concentrations in *Salvinia rotundifolia* in different structures and found that 0.8 mg/g accumulated in fronds and 1.3 mg/g in laciniae, at pH 6 and a concentration of 20 mg/L Cr(III), conditions similar to those of our study [33].

In *E. crassipes*, BAF values for leaves were 37 ± 12 and 21 ± 6, while in roots, they were 1.2264 ± 35 and 326 ± 93 for concentrations of 2 and 20 mg/L of Cr(III), respectively. Increasing Cr(III) concentrations produced a decrease in BAF in leaves and roots (Table 2). Gardea-Torresdey et al. observed that at concentrations <5 mg/L of Cr(III), chemical hormesis occurs in *Salsola kali* [56]. Papadia et al. notice that Cr(III) is not as well translocated to the aerial part as other heavy metals (example: Cd(II) or As(III)) [57]. According to Ding et al., Cr(III) translocates little in *Arabidopsis thaliana* and slightly less than Cr(VI) [58]. TF values were determined for the above concentrations and yielded very low results. This indicates that the main mechanism of Cr(III) adsorption in *E. crassipes* is at the root level, with little involvement of the leaves or aerial parts. Hadad et al. explain that Cr(III) adsorption by the aerial parts of *E. crassipes* is negligible because its leaf morphology is different from that of other macrophytes, with translocation from roots to aerial parts being the only process responsible for Cr accumulation in leaves [54]. The concentration of Cr in water is the main factor that influences the efficiency of metal absorption by the plant. When the concentration of Cr in the water increases, the accumulation of the metal in the plant increases while the BAF decreases. Our study demonstrated that higher BAF values are achieved when the concentration of Cr(III) in a solution is low (2 mg/L) compared to higher concentrations (20 mg/L). The general comparison of both plants showed that *E. crassipes* is a better bioremediator than *S. auriculata*, as it outperforms the latter in terms of removal rate and tissue uptake. In a hydroponic system, a high BAF (≥1000) for whole plant tissue indicates the phytostabilization potential of plants for heavy metals [59,60]. Therefore, *E. crassipes* can be considered a Cr(III) bioaccumulator plant, considering that the maximum BAF value determined is well above 1000.

### 3.2. Chromium (III) Phytoremediation and Tannery Effluent Toxicity

The variation of pollutants in the effluent at 72 h of treatment is presented in Table 3. After the phytoremediation treatment, the BOD/COD ratio of the primary effluent increased from 0.69 to 0.89 for the sample treated with *S. auriculata* and to 0.88 for the sample treated with *E. crassipes*, evidencing a capacity to increase biodegradability for both plants. The BOD/COD ratio is an indicator of the degradability of organic matter. A ratio of >0.5 indicates that the organic matter is readily biodegradable, while a ratio of <0.3 means that the organic matter is difficult to degrade in wastewater treatment [61]. Mangkoedihardjo [62] demonstrated that *E. crassipes* can increase the biodegradability of industrial effluents between 0.3 and 0.5 BOD/COD; this behavior would be closely linked to the initial COD. For an initial COD of <500 mg/L, the rate of increase in the BOD/COD ratio was 1.5 times faster than for an initial COD of >500 mg/L. The effluent treated with *S. auriculata* showed a reduction in all parameters, and the same behavior was observed in the control. Meanwhile, the effluent treated with *E. crassipes* showed a slight increase in the concentration of N-ammonia (negative reduction of −12%) and orthophosphate (−1%). The control without macrophytes presented the best levels of N-ammonia removal (27%). N-ammonia removal can occur using different processes including volatilization, direct plant uptake, and microbial action. Volatilization is favored by high pH and the absence of coverage between the water–atmosphere interface [63]. Pinaffi and Santos [64] showed that the presence of the macrophytes *E. crassipes* and *S. auriculata* decreased the volatilization rate, which might explain the better removal observed in the control without macrophytes.

Regarding the low removal of organic matter, the best results were observed in *E. crassipes*, with values of 14% and 33% for BOD and COD, respectively. The COD removal was significantly higher for *E. crassipes* (*p* < 0.05) compared to *S. auriculata* and the control. The lower removal efficiencies in *S. auriculata* (1% and 23% for BOD and COD) for these compounds might be associated with the senescence observed in some plants at 72 h. Schwantes et al. found that the removal capacity of COD, volatile solids, and fixed solids decreased with hydraulic retention time in systems using *S. auriculata* for the treatment of dairy wastewater and associated it with the deterioration and/or death of the plants [44].

**Figure 3:**
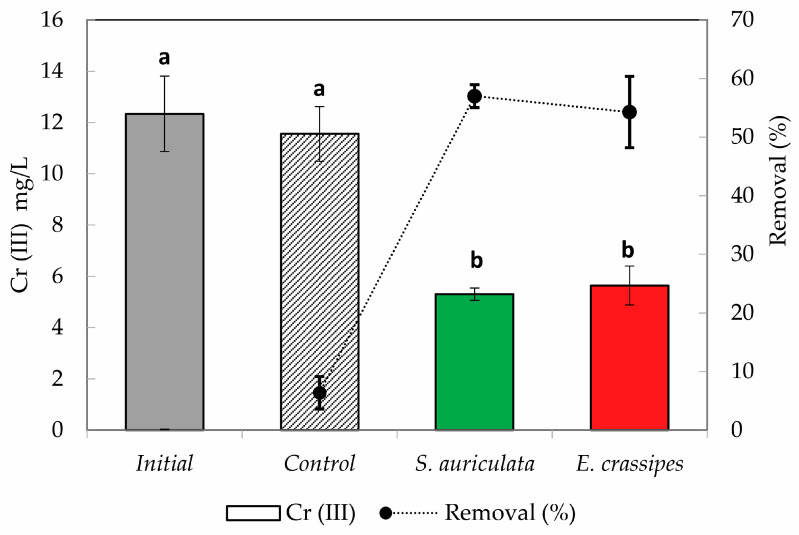
Cr(III) removal after phytoremediation treatment of tannery effluent. Each value is the mean of the three replicates, with bars representing standard deviations. Letters correspond to ANOVA and homogeneous subsets (*p* < 0.05).

Both plants showed significantly higher chromium removal capacity (*p* < 0.05) compared to the control (6%), with a 57% reduction for *S. auriculata* and 54% for *E. crassipes* for the conditions of the assay (Figure 3). Previous studies have demonstrated the ability of E. *crassipes* to concentrate and remove Cr(III) associated with industrial effluents. *E. crassipes* showed the ability to bioconcentrate chromium present in water, sediments, and soils highly contaminated by tannery effluent discharges in industrial areas of the Dhaleshwari River [17]. Meanwhile, Kassaye et al. demonstrated that several species, such as *Polygonum coccineum*, *Brachiara mutica*, and *Cyprus papyrus*, can remove Cr(III) from tannery effluents working with samples at low concentrations (1 mg/L), obtaining removal efficiencies of 51%, 31%, and 73%, respectively [14].

According to the Water Quality Standard of the Republic of Paraguay, the maximum discharge values for Cr(VI) and Cr(III) are 0.5 and 2 mg/L, respectively [49]. Usually, the regulations in different parts of the world require chromium concentrations below 0.5 mg/L in wastewater. However, in countries such as China, the maximum contaminant level (MCL) of total Cr in effluent discharges from the leather industry is set at 1.5 mg/L [65]. In our study, we found that the removal of Cr(III) in wetlands with *E. crassipes* and *S. auricualata* is not efficient enough to comply with the legislation when working with real effluents with initial concentrations > 12 mg/L of Cr(III). This behavior could be associated with the remaining toxicity inherent to these types of wastewater.

### 3.3. Toxicity Assays

#### 3.3.1. Toxicity Assay with *Daphnia magna*

**Figure 4:**
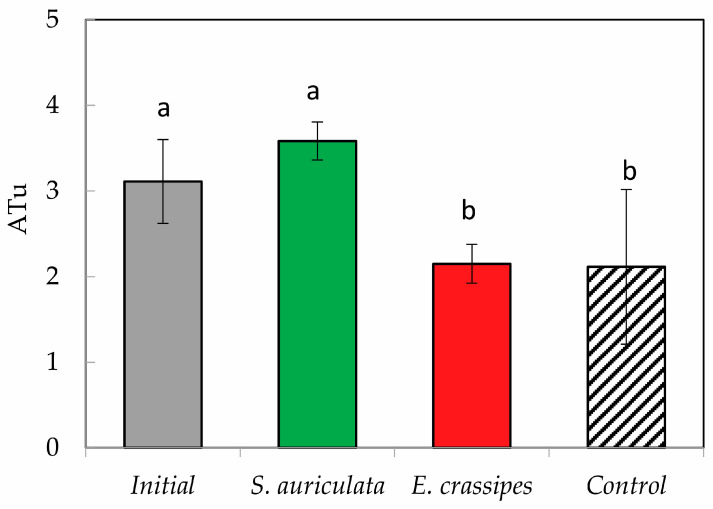
Variation of acute toxicity in *Daphnia magna* in the different treatment of tannery effluent. Each value is the mean of the three replicates, with bars representing standard deviations. Letters correspond to ANOVA and homogeneous subsets (*p* < 0.05).

To calculate the LC_50_, the *D. magna* that showed total immobility observed under the microscope (10×) was counted after 48 h of exposure to the treatments. The acute toxicity assay on *D. magna* showed a reduction of 0.96 ATu (*p* < 0.05) for neonates exposed to an *E. crassipes*-treated effluent (2.15 ± 0.23 ATu) compared to the initial effluent (3.11 ± 0.49) (Figure 4). The effluent treated with *S. auriculata* did not produce a reduction in acute toxicity (3.58 ± 0.22 ATu). De Nicola et al. compared the toxicity of untreated wastewater from a traditional tannery (TTE) and a chromium tannery (CTE) on *D. magna* and found that CTE produced higher toxic effects close to 50% at dilutions of 3.2%, and 100% immobilization at dilutions of 12.5% [40]. The acute toxicity measured in our study is lower than has been reported by other researchers. One possible explanation might be related to the fact that we are dealing with primary effluents and not untreated effluents, which results in lower starting concentrations of toxicants and a high BOD/COD ratio.

Considering the physicochemical parameters in Table 2, high COD, BOD, and N-ammonia values are observed, which could be related to the remaining toxicity. The remaining toxicity of effluents treated via a biological process in *D. magna* and *D. pulex* is mainly related to ammonia and TDS [66].

#### 3.3.2. Toxicity Assays with *Danio rerio*

**Figure 5:**
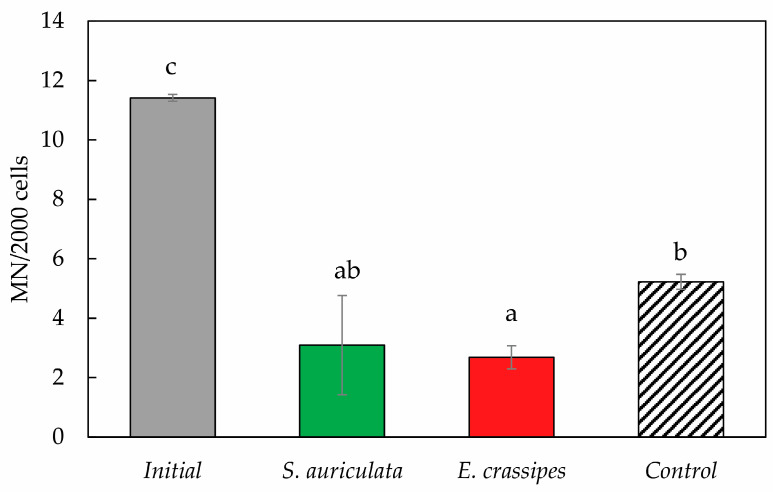
Frequency of micronuclei in 2000 *Danio rerio* cells in different treatments. Each value is the mean of the three replicates, with bars representing standard deviations. Letters correspond to ANOVA and homogeneous subsets (*p* < 0.05).

As shown in Figure 5, the frequency of micronuclei in peripheral blood erythrocytes of *Danio rerio* treated with *S. auriculata* and *E. crassipes* decreased significantly when compared to the initial effluent and the control. The control in Figure 4 and Figure 5 is the tannery effluent without the macrophytes. Micronuclei in erythrocytes are considered markers of mutagenicity caused by pollutants [67]. Low concentrations of chromium in tannery effluents (0.1–1.27 mg/L) produce detectable amounts of nuclear aberrations such as micronuclei in Nile tilapia (*Oreochromis niloticus*), where the most sensitive cells are gill cells > peripheral erythrocytes > kidney [10]. Cr(III) used to be considered relatively non-toxic, but in recent years, several studies have provided evidence that Cr(III) causes genotoxic damage [3,68]. The Cr(III) genotoxicity mechanism is related to interference with DNA base-pair stacking, which causes DNA cleavage and degradation. In a controversial study, Fang et al. concluded that Cr(III) produces mutations in yeasts and Jurkat cells, i.e., in eukaryotic models [4]. The cause of the presence of micronuclei in *D. rerio* cannot be attributed solely to the presence of chromium. Tannery effluents are known to be complex since over 150 organic and inorganic compounds are used in the tanning process [8]. Chagas et al. demonstrated that even highly diluted tannery effluents (0.1 and 0.3%) induce behavioral changes in adult zebrafish, suggesting a neurotoxic effect [38].

## 4. Conclusions

It was proven that *E. crassipes* and *S. auriculata* could efficiently reduce the Cr(III) content in hydroponic solution. *E*. *crassipes* presented higher bioaccumulation capacity in tissues, with a BAF 1334 times higher than *S. auriculata* when exposed to a concentration of 20 mg/L Cr(III) in solution. However, both plants show a decrease in removal efficiency with an increasing metal concentration in the solution.

In the experiments with primary tannery effluent, *E. crassipes* was more efficient in removing COD from wastewater. Both plants removed over 50% of Cr(III) in the effluent, although with lower performance compared to the hydroponic solution experiments, i.e., the removal capacity decreases when the plants are exposed to real tannery effluents. The efficiency of Cr(III) removal from real tannery effluents when working with metal concentrations > 12 mg/L does not allow compliance with local legislation requiring Cr(III) levels < 2 mg/L, which would imply the need for other previous or subsequent treatments.

Toxicity tests using *D. magna* showed that *E. crassipes* has the ability to reduce the acute toxicity of tannery effluent. However, this ability was not observed in effluents treated with *S. auriculata*. The micronucleus test in the peripheral blood of *D. rerio* revealed significant removal of the genotoxicity of the effluent for both plants.

## Figures and Tables

**Table 1:** Final Cr(III) concentrations in plant tissues and bioaccumulation factor (BAF) in dry biomass. Each value is the mean of the three replicates ± standard deviation.

Macrophyte	Cr(III) Treatment (2 mg/L)		Cr(III) Treatment (20 mg/L)	
	mgCr/Kg Dry Weight	BAF	mgCr/Kg Dry Weight	BAF
*S. auriculata*	0.33 ± 0.03	0.28 ± 0.26	5.24 ± 0.37	0.26 ± 0.02
*E. crassipes*	2734 ± 34	1302 ± 16	8159 ± 1.448	347 ± 62

**Table 2:** Final Cr(III) concentrations in *E. crassipes* tissues, BAF, and TF in dry biomass. Each value is the mean of the three replicates ± standard deviation.

*E. crassipes*	Cr(III) Treatment (2 mg/L)			Cr(III) Treatment (20 mg/L)		
	mgCr/Kg Dry Weight	BAF	TF	mgCr/Kg Dry Weight	BAF	TF
Leaf	78 ± 25	37.29 ± 12.2	0.029 ± 0.01	498 ± 137	21.19 ± 5.87	0.019 ± 0.012
Root	2656 ± 73	1264 ± 34.81		7660 ± 2185	326 ± 93.12	

**Table 3:** Physicochemical characteristics and removal values after phytoremediation treatment of tannery effluent.

Parameters	*S. auriculata*	*E. crassipes*	Control
BOD, mg O_2_/L	450 ± 0.58 (1%)	390 ± 0.58 (14%)	440 ± 34.64 (4%)
COD, mgO_2_/L	503 ± 16.71 ^a^ (23%)	443 ± 17.57 ^b^ (33%)	508 ± 2.1 ^a^ (23%)
BOD/COD	0.89 ± 0.03 (29%)	0.88 ± 0.04 (27%)	0.86 ± 0.06 (24%)
Total Phosphorus (P), mg/L	0.55 ± 0.1 (15%)	0.66 ± 0.02 (−1%)	0.47 ± 0.01 (28%)
N-Ammonia (N-NH4),mg/L	53.6 ± 11.4 ^ab^ (11%)	67.55 ± 2.23 ^a^ (−12%)	43.86 ± 8.39 ^b^ (27%)
TKN (N), mg/L	63.85 ± 4.74 (23%)	77.69 ± 8.59 (6%)	64.83 ± 12.07 (21%)
Sulfides (S^−2^), mg/L	1.35 ± 0.21 (94%)	1.3 ± 0.26 (94%)	1.05 ± 0.07 (95%)

The mean concentration of the three replicates and the standard deviation for each physicochemical parameter are presented. The value presented in parentheses corresponds to the removal of each parameter. The letters (^a,b^) correspond to the ANOVA and the homogeneous subsets (COD and N-Amnonia). BOD, BOD/COD, total phosphorus, TKN, and sulfides do not present significant differences (ANOVA and Kruskal–Wallis test).

## Data Availability

Data are contained within the article.
